# A Single-Nucleotide Polymorphism in an Endo-1,4-β-Glucanase Gene Controls Seed Coat Permeability in Soybean

**DOI:** 10.1371/journal.pone.0128527

**Published:** 2015-06-03

**Authors:** Seong-Jin Jang, Masako Sato, Kei Sato, Yutaka Jitsuyama, Kaien Fujino, Haruhide Mori, Ryoji Takahashi, Eduardo R. Benitez, Baohui Liu, Tetsuya Yamada, Jun Abe

**Affiliations:** 1 Research Faculty of Agriculture, Hokkaido University, Sapporo, Hokkaido 060-8589, Japan; 2 National Institute of Crop Science, 2-1-18 Kannondai, Tsukuba, Ibaraki, 305-8518, Japan; 3 Northeast Insititute of Geography and Agroecology, Chinese Academy of Sciences, 138 Haping Road, Harbin 150040, China; Southern Crop Protection and Food Research Centre, CANADA

## Abstract

Physical dormancy, a structural feature of the seed coat known as hard seededness, is an important characteristic for adaptation of plants against unstable and unpredictable environments. To dissect the molecular basis of *qHS1*, a quantitative trait locus for hard seededness in soybean (*Glycine max* (L) Merr.), we developed a near-isogenic line (NIL) of a permeable (soft-seeded) cultivar, Tachinagaha, containing a hard-seed allele from wild soybean (*G*. *soja*) introduced by successive backcrossings. The hard-seed allele made the seed coat of Tachinagaha more rigid by increasing the amount of β-1,4-glucans in the outer layer of palisade cells of the seed coat on the dorsal side of seeds, known to be a point of entrance of water. Fine-mapping and subsequent expression and sequencing analyses revealed that *qHS1* encodes an endo-1,4-β-glucanase. A single-nucleotide polymorphism (SNP) introduced an amino acid substitution in a substrate-binding cleft of the enzyme, possibly reducing or eliminating its affinity for substrates in permeable cultivars. Introduction of the genomic region of *qHS1* from the impermeable (hard-seeded) NIL into the permeable cultivar Kariyutaka resulted in accumulation of β-1,4-glucan in the outer layer of palisade cells and production of hard seeds. The SNP allele found in the NIL was further associated with the occurrence of hard seeds in soybean cultivars of various origins. The findings of this and previous studies may indicate that *qHS1* is involved in the accumulation of β-1,4-glucan derivatives such as xyloglucan and/or β-(1,3)(1,4)-glucan that reinforce the impermeability of seed coats in soybean.

## Introduction

Physical dormancy is present in at least 15 families of angiosperms [1]. It is attributed to a structural feature of the seed coat called ‘hard seededness’, which physically regulates the penetration of water into the seed. Hard seededness is an adaptive trait for wild plants that not only extends seed longevity [2] and persistence in soil seed banks [3], but also protects against microbial attack [4] and escapes the predation from scatter-hoarding rodents that detect seeds by olfaction [5]. Hard seededness is usually associated with the presence of one or more layers of impermeable palisade cells; structural features controlling water permeability in seed coats vary with plant taxa [1]. Despite its adaptive significance, the molecular basis of physical dormancy is not well understood.

Soybean (*Glycine max* (L.) Merr.) is an important legume crop that represents a major source of protein and vegetable oil supplements for humans and livestock worldwide. It is widely accepted that soybean was domesticated from a wild progenitor, *G*. *soja*, in the eastern half of north China, and then disseminated to various regions of Asia [6]. Cultivated and wild soybeans differ in a set of various morphological and physiological characteristics collectively designated as the domestication syndrome [7–9]. The typical cultivated phenotype displays a bush-type growth habit with a stout primary stem and sparse branches, bearing large seeds with variable seed coat colors, whereas the wild phenotype is a procumbent or climbing vine with a slender, many-branched stem bearing small, coarse black seeds. The wild soybean also differs in the extent of hard seededness from the cultivated soybean, although genetic variation exists in the latter for this trait [10–14].

Hard seededness in the cultivated soybean is typically associated with various functions [15]. It is implicated in seed viability under delayed-harvest field condition [16], seed longevity under humid environments [17, 18], tolerance to fungal activity [19–21], and inhibition against rapid imbibition of water, which often deteriorates the germination [22, 23]. On the other hand, seeds with impermeable seed coats, so-called stone seeds, often result in adverse quality and cost factors in processing seeds for vegetable oil and soy foods, and they affect the texture and consistency of products such as fermented soy food [24–27].

Several studies have investigated the mechanism of hard seededness in soybean from morphological and biochemical points of view [14, 26–31]. Morphological observation has revealed that hard seededness can be attributed to absence or scarcity of minute cracks [27] and the presence of a prominent light line in subcuticular layer [10]. In the seed coat of permeable cultivars, Ma et al. (2004) found numerous minute cracks on the dorsal side, the area through which water first enters the seed [32, 33], whereas the cuticle of an impermeable seed coat is mechanically strong and does not crack under normal conditions [27]. They assumed that the cuticle of the palisade layer is the key factor that determines the permeability of seed coats. Other authors have proposed that the compositions of carbohydrates, hydroxylated fatty acids or phenol compounds in seed coats control the level of permeability [14, 26, 28, 30]. Mullin and Xu (2001) found that the seed coat of an impermeable experimental line, OX951, had a high concentration of hemicellulose, essentially composed of xylans, which would reduce the hydrophilicity of the seed coat and increase stone seed production [26]. On the other hand, Shao et al. (2007) found that the cuticle of the impermeable line contained a higher amount of hydroxylated fatty acids than those of permeable cultivars, implying that the presence of a greater proportion of hydroxylated fatty acids may provide a greater interconnectivity between monomers in the cutin of hard seeds [28]. Saio (1976) also reported that the coats of impermeable seeds contained a high amount of calcium relative to those of permeable seeds [24]. All of these factors may influence seed coat structure, but it remains undetermined which of these factors are related to the genetic variation observed in seed coat permeability.

The genetic control of hard seededness in soybean has also been studied in crosses between cultivated and semi-wild or wild soybeans [9, 34–38]. These studies have indicated the involvement of several genes and/or quantitative trait loci (QTLs) with different gene actions. QTL analyses have further revealed genomic positions at which genes for impermeability are located. Of the QTLs that have been reported so far, a QTL located in linkage group (LG) D1b (chromosome 2), *qHS1*, is common across different cross combinations; this QTL also has the greatest effect on impermeability [9, 34–36, 38].

To dissect the molecular basis of hard seededness, we developed a near-isogenic line (NIL) of a permeable cultivar, Tachinagaha, containing a hard-seed allele at *qHS1* from a wild soybean accession. Since Tachinagaha is an easily imbibed cultivar with numerous minute cracks on the seed coat surface [27, 28], the introgression of the hard-seed allele into the Tachinagaha background facilitated the molecular analysis of *qHS1* and characterization of its role in seed coat structure. Here, we report that *qHS1* encodes an endo-1,4-β-glucanase, which makes seed coats more rigid and adaptable to dehydration stress during maturation by producing β-1,4-glucan derivatives on the outer layer of palisade cells and reinforcing the seed coat impermeability in soybean.

## Materials and Methods

### Plant Materials

A B_5_F_3_ family (#96-3-1) segregating for seed coat permeability (SCP) was used for positional cloning of *qHS1* and characterization of coat morphology of hard seeds. This family was developed from successive backcrossings of a cross between Japanese soybean cultivar Tachinagaha (TA) and a wild soybean plant collected in Aomori Prefecture, Japan (COL/AOMORI/1983NASU-2; AO), in which the former was used as the recurrent parent. The wild accession was obtained from the NIAS (National Institute of Agrobiological Science) Genebank, Japan. A survey of 80 SSR markers, comprising four markers selected from each of the 20 linkage groups, revealed that the family possessed the AO allele at only one SSR marker (Satt172), which is separated by 8 cM in a consensus map [39] from Satt459, previously identified as a tagging marker for *qHS1* [9]. The progeny (B_5_F_4_) of a single plant homozygous for the *qHS1* region was then used as a NIL for the hard-seed allele, and designated as TA-HS. A total of 194 cultivated accessions introduced from various Asian countries were used to test for an association between DNA polymorphism in *qHS1* and SCP. SCP was defined as the percentage of hard seeds that had not imbibed water after soaking for 6 h at room temperature.

### Fine-Mapping

A total of 665 seeds produced by three sibs of TA-HS heterozygous at Satt459 were genotyped with four public and eight newly designed flanking SSR markers. To develop the eight markers, we first searched for SSRs with more than 10 repeats of the AT motif in the genomic sequence of Williams 82 (http://www.phytozome.net/), and designed primers to amplify a fragment of ca. 150 to 200 bp encompassing the SSR. DNA extraction from seeds and SSR analysis followed the method of Xia et al. (2012) [40]. Of seeds having recombination among the markers, seven had combinations of markers homozygous for the TA allele and heterozygous for the TA and AO alleles; these were used for delineating the position of *qHS1*. SCP for each of the seven recombinants was evaluated by a progeny test; the percentage of hard seed was calculated by scoring a total of 40 seeds (two replications with 20 seeds each) from each of 10 to 15 plants. The association between the markers and SCP was further confirmed by testing the SCP of plants homozygous for recombinant genotypes in the region of interest. The primers used for the fine-mapping are listed in S1 Table.

### Expression and Sequence Analysis

Seed coats, and immature cotyledons as comparison, were sampled from developing immature seeds of TA and TA-HS at reproductive stage R6, when the pod reaches its maximum size [41]. All tissues were immediately frozen in liquid N_2_ and stored at -80°C. Total RNA was isolated from frozen tissues following the lithium chloride precipitation procedure [42], except that we removed genomic DNA from the RNA fraction using DNase I (Takara Bio, Kyoto, Japan). Methods for the purification of mRNA and synthesis of cDNA have been described in detail [43]. Real-time PCR was performed using the cDNA as a template to search for genes expressed in seed coats from among the genes annotated in the genomic region delimited by fine-mapping. The transcripts covering the entire coding regions of genes expressed in seed coats were then amplified by using the cDNA as a template, and the amplified products were cloned and sequenced. We used the DNA Sequencing Facility of the Research School of Agriculture, Hokkaido University. The predicted amino acid sequences were aligned using the ClustalW Multiple Sequence Alignment program version 1.8 (http://clustalw.genome.jp; [44]). The primers used for the expression analysis, cloning, and sequencing are listed in S2 and S3 Tables.

### Transformation

We transformed a soybean cultivar with permeable seed coats, Kariyutaka (KA), with a binary vector including the genomic region containing the AO allele at *qHS1*, using *Agrobacterium tumefaciens* strain EHA105 according to the method of Sato et al. (2007) [45]. To construct the vector, a 6,273-bp region containing the putative promoter and coding region of *qHS1* was amplified from the genomic DNA of TA-HS using primers 5′-AGCAAGCTTAGAGGATTAAACAATTCAAAC-3′ and 5′ -GGCAAGCTTGCCCCCTGATTCTTGGCGTTCAAGT-3′. The PCR was performed with KOD-Plus-Neo (Toyobo, Osaka, Japan) using the manufacturer’s instructions. The amplified fragment was purified with an Amicon Ultra centrifugal filter (Sigma-Aldrich, St. Louis, Missouri, USA). After excision by *Hin*dIII digestion, the fragment containing the genomic region of *qHS1* was cloned into the site between the *GFP* and *bar* (phosphinothricin resistance gene) cassettes in binary vector pMDC123-GFP, and the sequence was confirmed. A T2 line homozygous for the *GFP* transgene (KA-GFP), generated by transformation with the empty pMDC123-GFP vector, was used as a negative control. Transformed KA plants (KA-qHS1) were grown in a growth room with a constant air temperature of 23°C and average photon flux of 270 μmol photons m^−2^ s^−1^ with a daylength of 16 h.

### DNA Polymorphism Analysis and Association Test

An association test was performed between SCP and a functional DNA polymorphism detected between TA and TA-HS. The SNP was analyzed with a cleaved amplified polymorphic sequence (CAPS) marker, as follows. PCR using primers 5′-AATCTCTGGTACCCTCCCAT-3′ and 5′-TGTCCTAAAGACAAGACAGCA-3′ amplified a 443-bp fragment from the genomic DNA of both TA and TA-HS. The amplified fragment from the TA-HS allele has a *Pvu*II site (CAGCTG) containing the SNP; thus it is digested into 294- and 149-bp fragments with this enzyme whereas the TA allele remains undigested. To perform the association test, 10 μL of each PCR product was treated with *Pvu*II overnight, separated by electrophoresis on a 1% agarose gel, and visualized with ethidium bromide under UV light. The CAPS analysis was carried out for 69 seedlings derived from hard seeds detected in 28 accessions.

### Scanning Electron Microscopy (SEM) Analysis

Surface features of dry seed coats with and without cuticle were examined. Samples (approx. 25 mm^2^) were excised from the dorsal side of seeds, which is reported to be the area of first entrance of water into the seed coat [27, 32, 33]. Five seeds were examined for each line. Cuticles were removed from the seed coat surface by immersing seeds in hot (60°C) 1 M NaOH for 5 min. Then the samples were dehydrated in an ethyl alcohol series (50–100%) and were completely dried with a critical-point dryer (HCP-2, Hitachi Ltd., Tokyo, Japan). The dried samples were mounted on the metal stage of a SEM and coated with platinum particles by using an ion sputter (E101, Hitachi Ltd., Tokyo, Japan). All samples were observed with a SEM (JSM-5310LV, JEOL Co., Tokyo, Japan) at 15 kV.

### Detection of β-1,4-Glucan in Cross-Sections of Seed Coats

Seed coats from the dorsal side of seeds were embedded into 5% agar and then sliced into sections (30-μm) with a microslicer (DTK-1000, Dosaka, Kyoto, Japan). The sliced pieces were stained with 1 mg/L calcofluor white M2R (Polysciences, Inc., Warrington, PA) solution and examined under UV illumination by confocal microscopy (Leica TCS-SP5, Leica Microsystems, Tokyo, Japan). The intensity of fluorescence was measured with the Leica TCS-SP5 program (Ver. 2.0), and averaged at the same relative positions along the long axis of the palisade cells to compare the intensity between TA and TA-HS or between KA-GFP and KA-qHS1.

## Results

### Development of the NIL for Hard Seededness

Takahashi and colleagues developed a backcross inbred population from a cross between Japanese cultivar Tachinagaha (TA) and a wild accession of *G*. *soja* collected in Aomori Prefecture, Japan. A NIL of TA containing the hard-seed allele from the wild accession (TA-HS) was developed from the progeny of a single plant homozygous for the *qHS1* region selected from a family segregating for hard seededness (#96-3-1). TA-HS produced seeds with yellow seed coat and yellow hilum (*I*/*I* and *t*/*t*, respectively) and almost the same size as those of TA (32 g/100 seeds for TA and 30 g/100 seeds for TA-HS). On an individual-plant basis, TA-HS produced averages of 46.7% (range 15–96%) and 29.2% (range 10–50%) of impermeable (hard) seeds at 6 h and 24 h after immersion, respectively. The ratio of seed coat weight to whole-seed weight was slightly but significantly higher in TA-HS (6.75%) than in TA (6.05%) (*t* = 8.03, *p* < 0.001).

### Seed Coat Structure of the NIL for Hard Seededness

**Fig 1:**
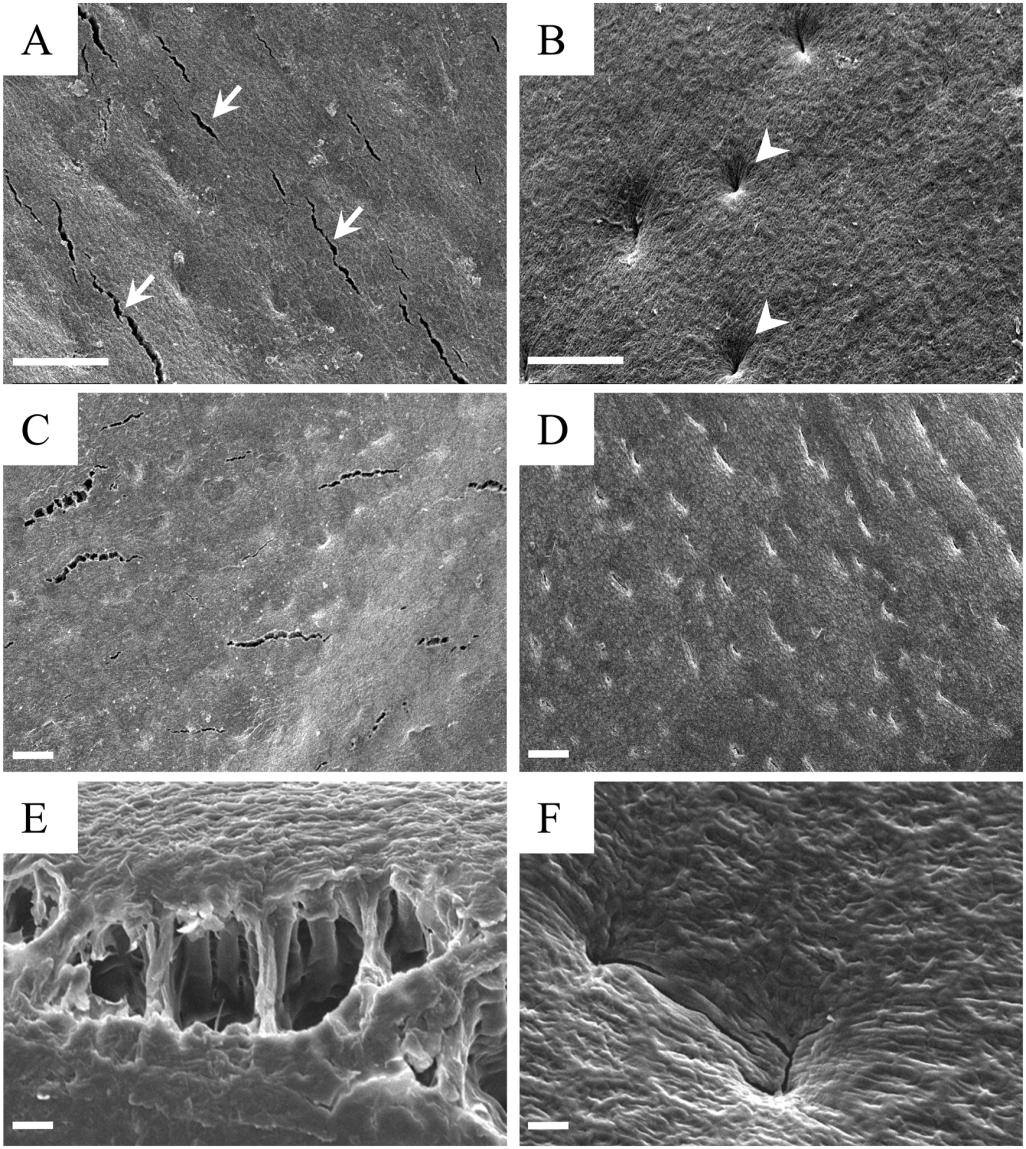
Surface features of seed coats in water-permeable cultivar Tachinagaha and its impermeable NIL. The structures of seed coats on the dorsal side of seeds were observed with SEM. Cracks (arrows) and pits (arrowheads) were observed in Tachinagaha (A) and its impermeable NIL (TA-HS) (B), respectively. C to F, Surface features after removing the cuticle with hot (60°C) 1 M NaOH. The cracks observed in Tachinagaha show a ladder-like structure (C and E) in which palisade cells are partly connected (E). Pits in TA-HS are closed (D and F). Bars in A to D = 50 μm; E and F = 5 μm.

**Fig 2:**
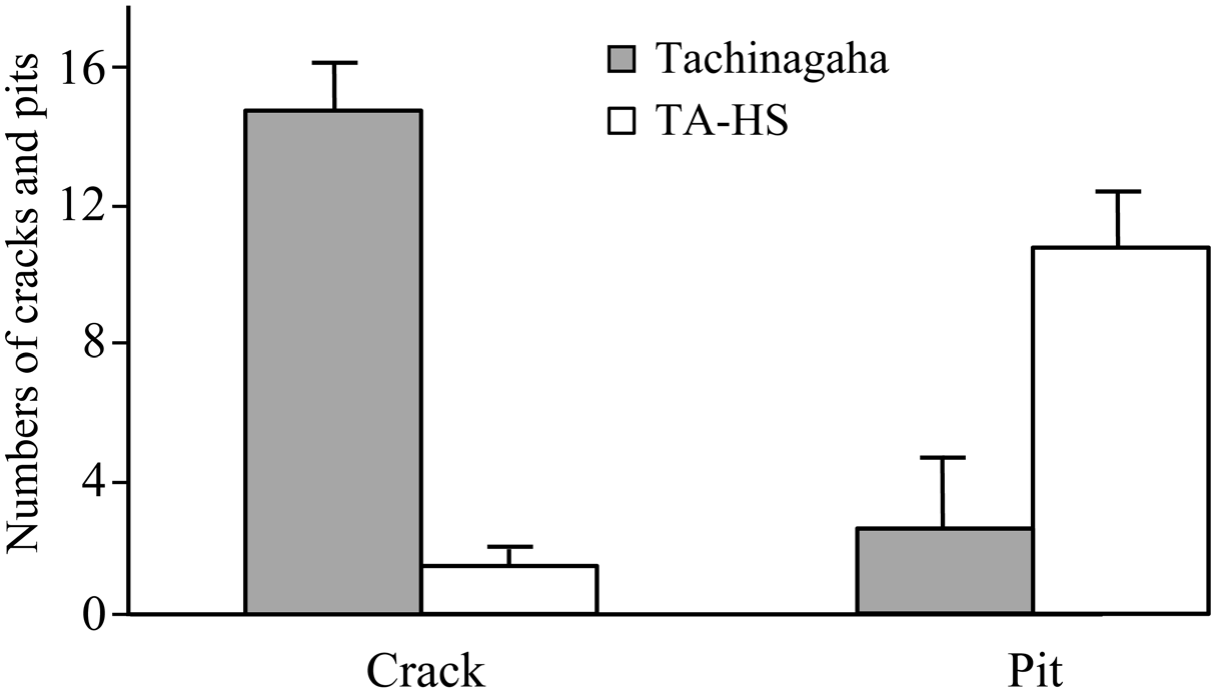
Frequencies of cracks and pits on seed coats. The numbers of cracks and pits per area (505 × 675 μm) in seed coats on the dorsal sides of seeds were significantly different (*p* < 0.001) between water-permeable cultivar Tachinagaha and its impermeable NIL (TA-HS)(*n* = 40). Error bars indicate the maximum errors of estimates at 95%.

The high moisture permeability of the seed coat of TA is ascribed to the occurrence of many minute cracks (1–5 μm wide and 20–200 μm long), present mainly on the seed coat of the dorsal side of seeds [27]. We also observed those cracks in the seed coat of TA (Fig 1A). However, such cracks were rarely observed in the impermeable seeds of TA-HS, which instead possessed small pits on the seed coat surface (Fig 1B). The number of cracks per unit area (505 μm × 675 μm) was significantly higher in TA than in TA-HS (*t* = 18.6, *p* < 0.001) (Fig 2). In contrast, the frequency of pits was significantly higher in TA-HS than in TA (*t* = 6.9, *p* < 0.001).

Ma et al. (2004) found that the cracks in permeable seeds typically extended through the cuticle into the periclinal walls of palisade cells, and a few extended deep into the palisade layer or even the cell layers underneath [27]. We observed the structures of underlying palisade layers by removing the cuticle from seed coats by a hot NaOH treatment. Cracks were observed in the surface of palisade layers of seeds from TA (Fig 1C). They exhibited a ladder-like structure, i.e., the neighboring palisade cells were not completely separated, but some remained in contact with each other (Fig 1E). In contrast, the surface of palisade layers in the impermeable seeds of TA-HS was smooth, with some shallow pores (Fig 1D). There was no separation of palisade cells: all of the pits were closed (Fig 1F).

### Fine-Mapping of a QTL for Hard Seededness, *qHS1*

We found that family #96-3-1 was segregating for seed coat permeability (SCP), which was associated with the genotype at Satt459, a marker used to tag *qHS1* [9]. We then selected three plants heterozygous for the AO (wild soybean) and TA alleles of Satt459 from this family for fine-mapping of *qHS1*. A total of 665 seeds produced by these plants were genotyped for 12 SSR markers. Four of these were public markers [39]; the others were newly designed based on the genomic sequence of Williams 82 [46]. A total of 18 recombinants were detected, of which seven had combinations of markers homozygous for the TA allele and heterozygous for the TA and AO alleles; these seven were used for more detailed genotyping of the *qHS1* region and phenotyping of the hard seededness trait.

**Fig 3:**
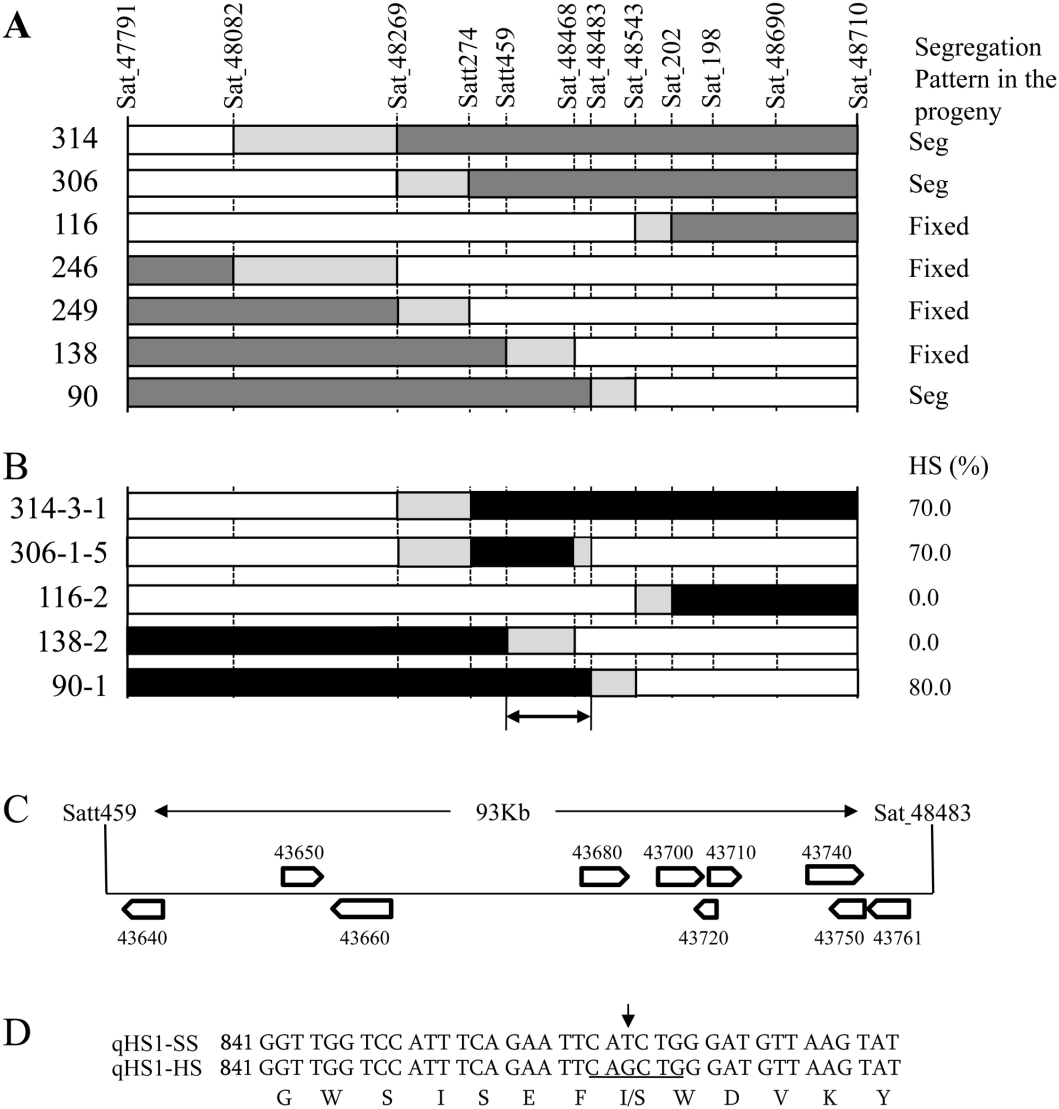
Fine-mapping of the QTL for hard seededness (*qHS1*). A segregating family of a backcross inbred line derived from a cross between Tachinagaha and a wild soybean accession was used for fine-mapping. Graphical genotypes are presented for plants (B_5_F_4_) with recombination in the genomic region harboring the QTL (A) and their progeny (B_5_F_6_) homozygous for recombinant genotypes (B). Open, dark gray, and black bars indicate regions homozygous for the allele from Tachinagaha (TA allele), heterozygous, and homozygous for the allele from the wild soybean (AO allele), respectively. Light gray bars indicate a region in which recombination occurred. Phenotypes for permeability are presented at right. In (A), the permeability of the B_5_F_4_ plants was evaluated based on the results of a progeny test (Seg, segregating for permeability; Fixed, homozygous for permeability). In (B), HS (%) indicates percent of hard seeds. C, The delineated region of 93 kb included 10 annotated genes in the Williams 82 genome sequence database (Phytozome Glyma1.0). D, Mutation site in the endo-1,4-β-glucanase gene (Glyma02g43680) and predicted amino acid sequences. Underline, *Pvu*II restriction site.

The genotype at *qHS1* for the seven plants was estimated based on the segregation pattern of SCP in the progeny. Three plants (#90, #306, and #314) that segregated for SCP in the progeny all possessed a heterozygous region between markers Satt274 and Sat 48483, whereas plant #138, which was homozygous for the TA allele of *qHS1*, was heterozygous for Satt459 but homozygous for the TA allele at Sat 48483 (Fig 3A). Accordingly, a candidate region of *qHS1* was delineated to the region from Satt459 to Sat 48483. Plants homozygous for recombinant regions selected from the progeny produced results similar to those of the progeny test (Fig 3B). In particular, an impermeable plant (#306-1-5) was homozygous for the AO alleles at Satt274 and Sat 48468, but homozygous for the TA allele at Sat 48483 (Fig 3B). Taken together, these data delineated *qHS1* to a genomic region of ca. 93 kb between Satt459 and Sat 48483. This region contained a total of 10 annotated genes in the Williams 82 genome sequence (Glyma1.0) (Fig 3C and S4 Table).

### Gene Expression and Sequencing

The ten annotated genes (S4 Table) were analyzed for their expression profiles in seed coats at the R6 stage, when immature seeds reach the maximum size [41]. Only two genes, Glyma02g43640 (endo-1,3- β-glucanase related) and Glyma02g43680 (endo-1,4- β-glucanase), were expressed in the seed coats of both TA and TA-HS (S1 Fig). No marked difference was observed in the transcript abundance of these two genes between TA and TA-HS. We then sequenced the cDNA of these two genes from TA and TA-HS. There was no difference in Glyma02g43640 sequences between the two lines, whereas a SNP was detected at the 863rd nucleotide from the adenine of the start codon in Glyma02g43680 (Fig 3D and S2 Fig). This gene was predicted to encode a protein of 524 amino acids (AAs), and the SNP caused an AA substitution from isoleucine (I) in TA to serine (S) in TA-HS (Fig 3D). The Williams 82 genome database (Glyma1.0) indicated that this cultivar has the same SNP allele as TA.

### Comparison of Amino Acid Sequences of *qHS1* and Other Plant Endo 1-4- β-Glucanase Genes

**Fig 4:**
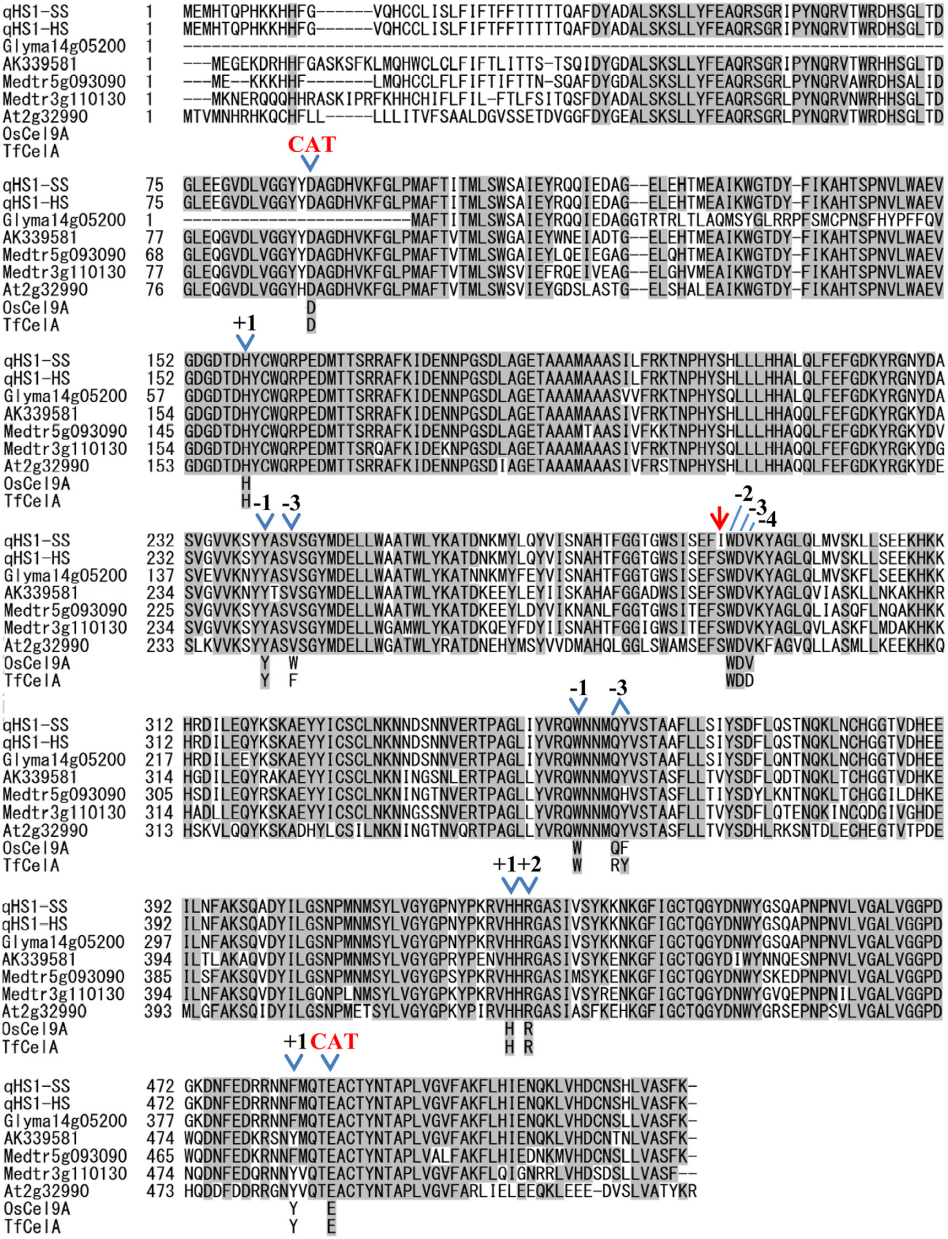
Amino acid sequence comparison among endo-1,4-β-glucanases from soybean and other plant species. The predicted amino acid sequences of *qHS1* and its homoeologous copy Glyma14g05200 were compared with predicted endo-1,4-β-glucanases of *Lotus japonicus* (AK339581), *Medicago truncatula* (Medtr5g093090 and Medtr3g110130), and *Arabidopsis thaliana* (At2g32990; AtGH9B8). The catalytic residues and amino acids involved in substrate binding of previously characterized glycoside hydrolase family 9 enzymes, OsCel9A, rice (*Oryza sativa* L.) endo-1,4-β-glucanase (Uniprot Q0JPJ1) and TfCel9A, *Thermobifida fusca* endo/exo-1,4-β-glucanase, are labeled with “Cat” and subsite numbers (+2 to -4), respectively. Arrow, substitution from isoleucine in Tachinagaha (*qHS1*-SS) to serine in TA-HS (*qHS1*-HS).

A survey of the Williams 82 genome sequence database using the predicted amino acid sequence of *qHS1* revealed that a homoeologous copy (Glyma14g05200) with an AA similarity of 89% existed in LG B2 (chromosome 14). The predicted AA sequence of Glyma14g05200 lacked the first 108 N-terminal AA of the sequence predicted from *qHS1* (Fig 4). The deduced AA sequence encoded by *qHS1* exhibited high AA similarities (80–83%) with predicted endo-1,4-β-glucanases of *Lotus japonicus* (AK339581) and *Medicago truncatula* (Medtr5g093090 and Medtr3g110130), and a similarity of 71% with that of *Arabidopsis thaliana* (At2g32990). Furthermore, comparison of the deduced AA sequences with those of previously characterized endo-1,4-β-glucanases from *Thermobifida fusca* (TfCelA), rice (*Oryza sativa*; OsCel9A), *Populus tremula × tremuloides* (PttCel9A), and *Populus alba* (PopCel1 and PopCel2) revealed that the soybean endo-1,4-β-glucanase protein encoded by *qHS1* contained the same AA residues at the catalytic site and most of the six substrate binding subsites (+2, +1, −1, −2, −3, and −4) (Fig 4 and S3 Fig).

Endo-1,4-β-glucanases belong to glycosyl hydrolase family 9 (GH9) [47], which is subdivided into three distinct structural subclasses: membrane-anchored (class A), secreted (class B), or secreted and having a carbohydrate binding module (class C) [48–50]. The *Arabidopsis* endo-1,4-β-glucanase gene At2g32990 encodes a class B endo-1,4-β-glucanase 11 (AtGH9B8) [50]. According to the standardized nomenclature by Urbanowicz et al. (2007) [50], we designated the endo-1,4-β-glucanase encoded by *qHS1* as GmGH9B8.

Comparison of the AA residue at which the nonsynonymous mutation was detected between TA and TA-HS further indicated that the serine residue found in TA-HS was common across Glyma14g05200 and the four proteins from *L*. *japonicus*, *M*. *truncatula*, and *A*. *thaliana*, and the isoleucine residue was specific to the permeable cultivars Tachinagaha (Fig 4). This AA residue is located next to the tryptophan residue (W) at substrate binding subsite −2 (Fig 4). The tryptophan residue is highly conserved (99.2%) across 971 endo-1,4-β-glucanase sequences that had an AA similarity of more than 70% to GmGH9B8 (NCBI; http://www.ncbi.nlm.nih.gov/). The isoleucine residue was detected only in a Williams 82 soybean sequence (XP_003519454.1); the most frequent AA was serine (61.3%), the second highest was glycine (29.8%), followed by asparagine (2.9%), alanine (2.6%) and threonine (1.8%). Thus, the AA substitution from serine to isoleucine that occurred in the substrate binding cleft may interfere with the affinity of the enzyme to substrates, modifying its function. Taken together, the data obtained in the present study suggest that *qHS1* (Glyma02g43680; Glyma.02g269400 in *Glycine max* Wm82.a2.v1) encodes an endo-1,4-β-glucanase (GmGH9B8) and that the detected SNP causes the difference in permeability between Tachinagaha and its impermeable NIL, TA-HS.

### Calcofluor White Staining in Cross-Sections of Seed Coats

**Fig 5:**
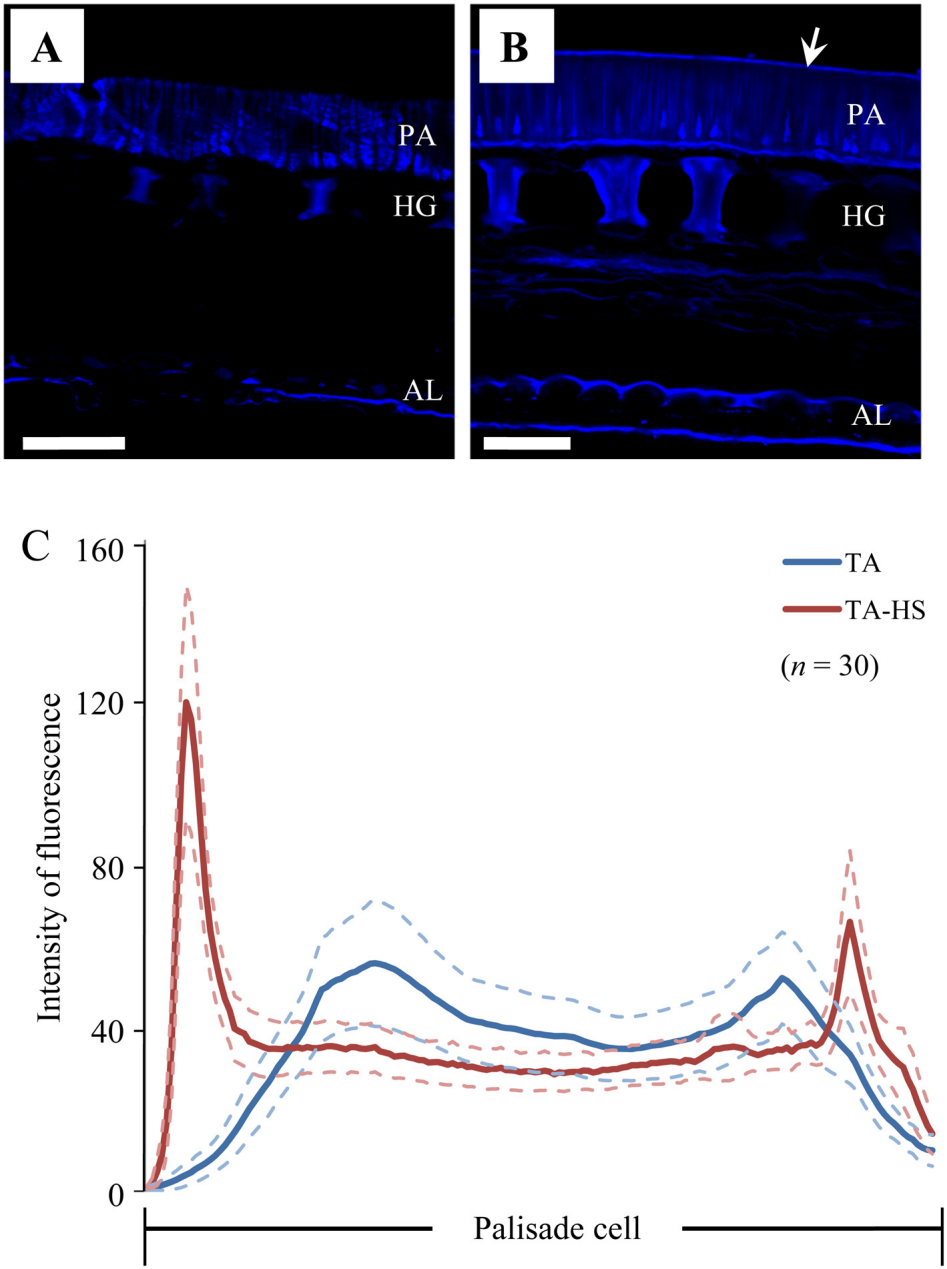
Calcofluor white staining in micro-sections of seed coat. Cross-sections of seed coats from the dorsal side of seeds of permeable cultivar Tachinagaha (A) and its impermeable NIL (TA-HS) (B) were stained with 0.1% calcofluor white solution and examined under UV illumination by confocal microscopy. Fluorescence indicates accumulation of β-1,4-glucan derivatives in palisade cells (PA), hourglass cells (HG), and outer and inner layers of the aleurone layer (AL). A fluorescent line (arrow) is observed in the outer layer of palisade cells in TA-HS (B), but not in Tachinagaha (A). C, Intensity of fluorescence averaged over 30 positions per genotype at the same relative positions along the long axis of the palisade cell. Blue and red lines are averages for TA-HS and Tachinagaha, respectively, with the maximum errors of estimates at 95% (light blue or pink dashed lines). Scale bars in A and B = 50 μm.

Plant endo-1,4-β-glucanases hydrolyze β-1,4-glucosyl linkages. We observed the accumulation of β-1,4-glucan in seed coats of TA and TA-HS by calcofluor white staining. Cross-sections of seed coats on the dorsal side of seeds were stained with calcofluor white and the fluorescence signals were observed under UV by confocal microscopy. The accumulation of β-1,4-glucan was observed in palisade cells, hourglass cells, and inner and outer layers of aleurone cells (Fig 5A and 5B). A consistent difference was found in the fluorescence intensity at the subcuticular layer between TA and TA-HS. The average intensity of fluorescence at relative positions along the long axis of the palisade cells was much higher at the outer layer in TA-HS than in TA (Fig 5C). Thus, the palisade cells of seed coats of TA-HS accumulated more β-1,4-glucan at the outer layers than those of TA.

### Transformation of the Functional *qHS1* Allele into a Permeable Cultivar

**Fig 6:**
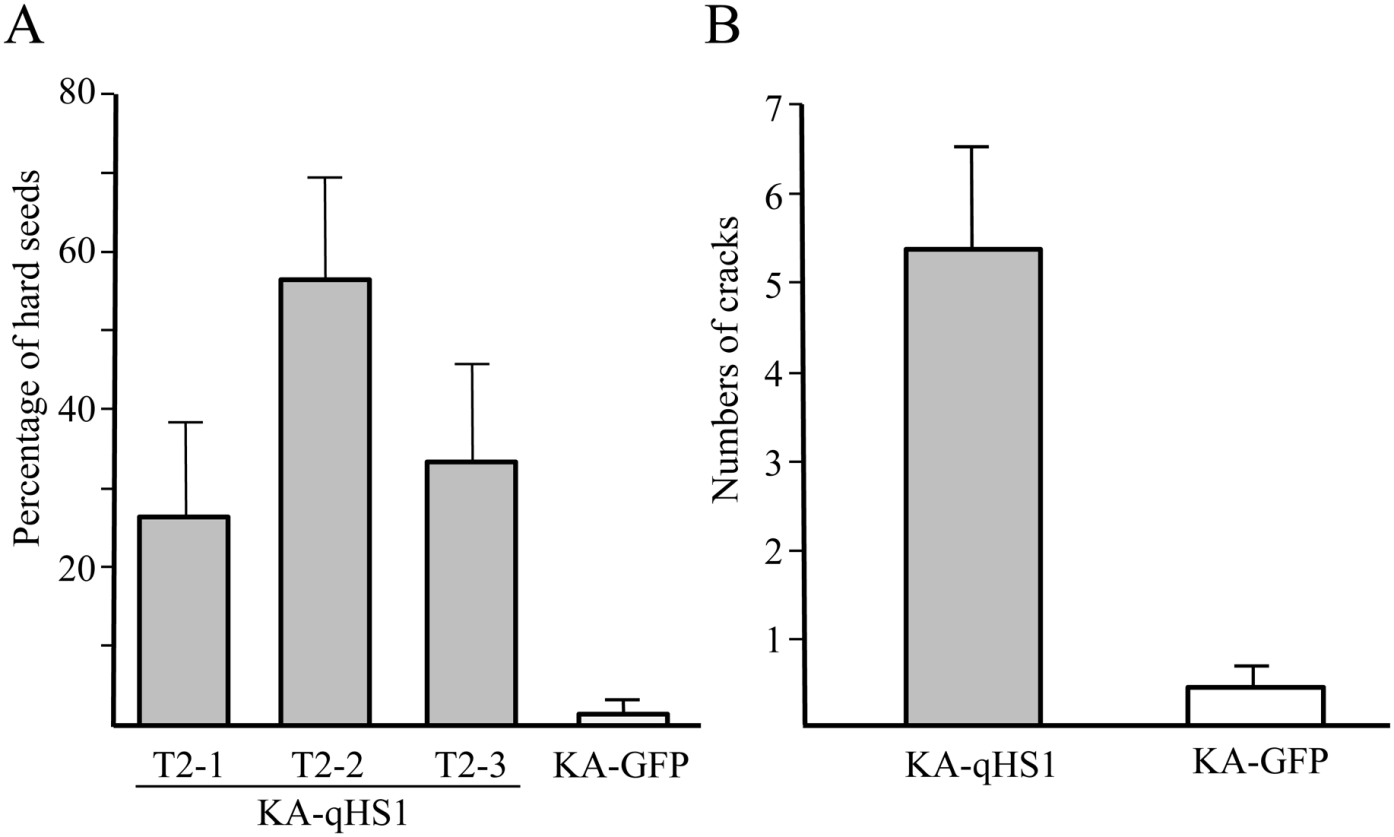
Seed coat characteristics of transgenic plants carrying the impermeable *qHS1* allele. A soybean cultivar with a permeable seed coat, Kariyutaka (KA), was transformed with the 6,273-bp genomic region containing the putative promoter and coding region of *qHS1* from TA-HS. T2 plants of three transgenic T1 plants (KA-qHS1; T2-1 to T2-3; gray bars) were compared with the T2 line transformed with the pMDC123-GFP construct (KA-GFP; open bars), which possessed only the GFP cassette. A, Average percentage of hard seeds (*n* = 60–100); B, Average number of cracks per area (270 × 361 μm) in seed coats on the dorsal side of seeds (*n* = 40). Error bars indicate the maximum errors of estimates at 95%.

To confirm that *qHS1* and Glyma02g43680 were identical, we transformed a soybean cultivar with a permeable seed coat, Kariyutaka (KA), with the 6,273-bp genomic region containing the promoter and 5′ untranslated region (UTR) sequences (1,903 bp), coding sequence (3,344 bp) and 3′ UTR and down-stream sequences (1,026 bp) from TA-HS. Expression of the green fluorescent protein (GFP) gene, located adjacent to the Glyma02g43680 transgene in the T-DNA region, was used as a marker for successful transformation. We analyzed the GFP-positive T2 progeny of three transgenic T1 plants (KA-qHS1), designated T2-1 to T2-3. A T2 line transformed with the pMDC123-GFP construct (KA-GFP), which possessed only the GFP cassette, was used for the negative control for evaluation of SCP. Most of the seeds produced by KA-GFP were permeable regardless of seed size, whereas the three KA-qHS1 T2 plants produced impermeable (hard) seeds at ratios of 25% to 57% (Fig 6A). Cracks similar to those in TA were also observed in seeds of KA-GFP, but rarely in those of the KA-qHS1 T2 plants (S4 Fig); the number of cracks was significantly higher in KA-GFP than in KA-qHS1 (*t* = 8.2, *p* < 0.001) (Fig 6B). Furthermore, the KA-qHS1 T2 plants produced seed coats with higher fluorescence intensity in the outer layer of palisade cells after staining with calcofluor white than did those of KA-GFP (S4 Fig). Therefore, the introduction of the Glyma02g43680 sequence from TA-HS into KA successfully reduced seed coat permeability by causing the accumulation of β-1,4-glucan in the outer layer of palisade cells.

### Association of a SNP in *qHS1* with Seed Coat Permeability

**Fig 7:**
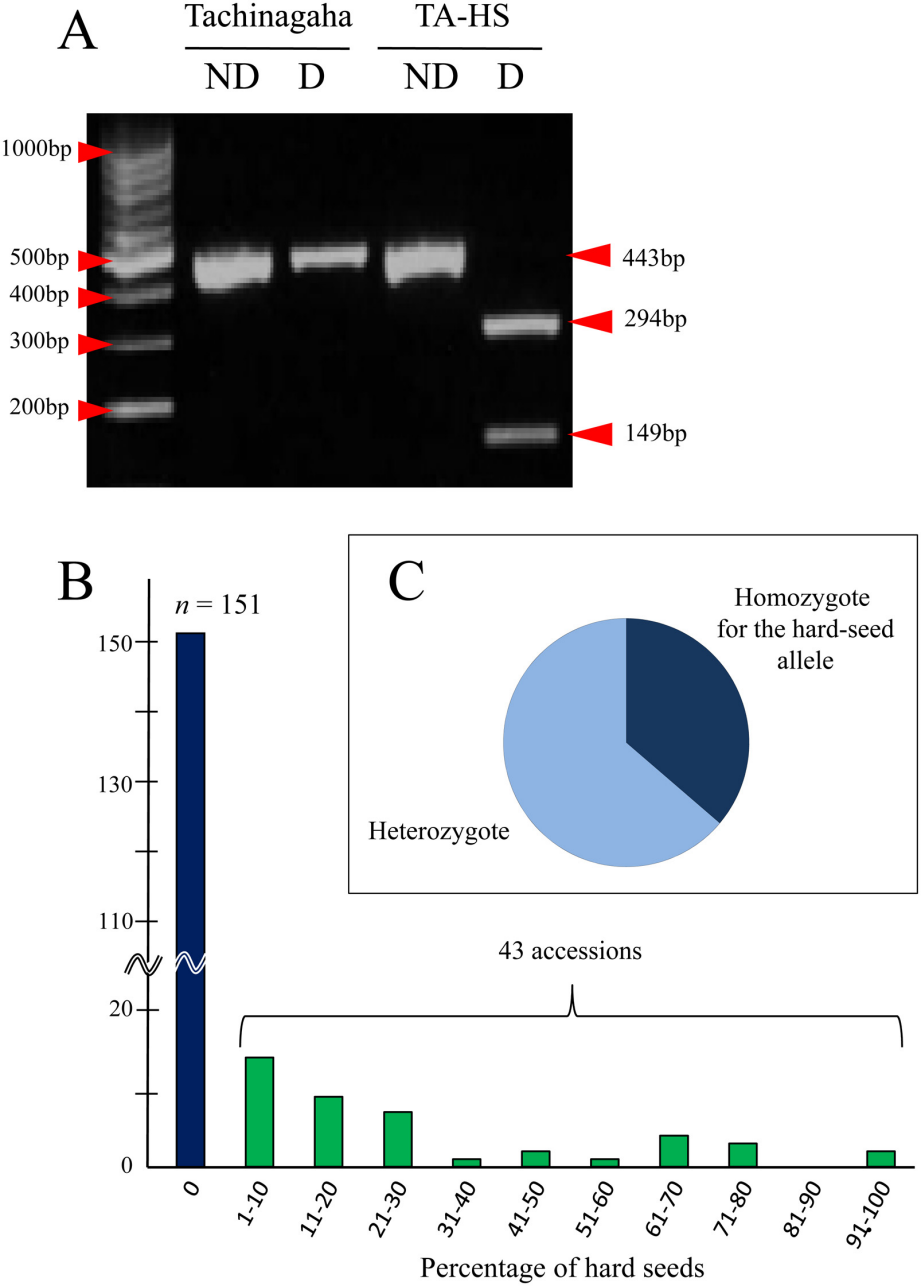
CAPS marker to detect the SNP in *qHS1*. A, A fragment of 443 bp harboring the SNP can be digested by *Pvu*II in the impermeable TA-HS, but not in the permeable Tachinagaha. ND, not digested; D, digested. M, Ladder marker (DNA-035, TOYOBO, Tokyo, Japan). B, Variation of seed coat permeability in 194 cultivated accessions. C, Genotypic constitutions at the CAPS marker for 69 impermeable seeds produced by 28 cultivated accessions.

Some soybean cultivars, particularly those with small seeds, have a tendency to produce hard seeds [25]. Impermeable soybean genotypes such as TA-HS also produce hard seeds at various ratios, depending on the environment during seed filling [51–54]. In order to determine to what extent the hard seeds produced in cultivars of different origins are accounted for by the SNP at *qHS1*, we tested the association between the SNP and production of hard seeds by using a CAPS marker. The sequence-specific PCR primers for this CAPS marker amplified a 443-bp fragment from the genomic DNA of both TA and TA-HS. The amplified fragment could be digested with *Pvu*II into 294-bp and 149-bp fragments in the latter, but not in the former (Figs 3D and 7A). The seed coat permeability was evaluated for a total of 194 small-seeded cultivated accessions with 100-seed weight of less than 20 g. Of these, 43 accessions produced impermeable seeds by varying ratios (Fig 7B). These included accessions from the Korean peninsula (7), China (13), Southeast Asian countries (14), and South Asia (9). Using the CAPS marker, we scored the genotype at the SNP of 69 impermeable seeds produced by 28 accessions; accessions that produced only one or two impermeable seeds were not included in the SNP analysis. All of the seeds tested possessed the nucleotide G (as did TA-HS) in either a homozygous or heterozygous condition (Fig 7C), suggesting that the impermeable seeds produced by those accessions may be determined by the genotype at *qHS1*.

## Discussion

### *qHS1* Encodes an Endo-1,4-β-Glucanase

Hard seededness, a determinant of physical dormancy in soybean, is one of the traits necessary for wild plants to adapt to unstable and unpredictable environments. To dissect the molecular basis of this trait, we developed a NIL of a permeable modern cultivar, Tachinagaha, containing a hard-seed allele at *qHS1*, which had the most common and marked effect on seed coat permeability among those that had been reported so far [9, 34–36, 38]. Tachinagaha had numerous minute cracks on the seed coat surface, whereas the seed coats of TA-HS did not have cracks (Fig 1); in addition, the seeds of TA-HS accumulated a higher amount of β-1,4-glucan on the outer layer of palisade cells on the dorsal side of the seeds (Fig 5). The introgression of a genomic region harboring *qHS1* into Tachinagaha was sufficient to confer hard seededness and make its fragile seed coat more resistant to cracking. The use of NILs for permeability thus enabled us to characterize in more detail the molecular and biochemical function of *qHS1* in seed coat permeability.

By fine-mapping and subsequent expression and sequencing analyses, we determined that *qHS1* encodes an endo-1,4-β-glucanase of 524 amino acids. The permeable cultivar Kariyutaka transformed with the genomic region of *qHS1* from the impermeable NIL produced impermeable seeds at ratios of 25% to 57% and formed fewer cracks than the GFP-only control (Fig 6A); this was also associated with the accumulation of more β-1,4-glucan derivatives in the outer layer of palisade cells (S4 Fig). Furthermore, the SNP responsible for different permeability of seed coats was tightly associated with occurrence of hard seeds in soybean cultivars of various origins. These results strongly suggest that the endo-1,4-β-glucanase gene is involved in the control of seed coat permeability in soybean.

### The Possible Role of *qHS1* in the Generation of Seed Coat Cracks

Endo-1,4-β-glucanases (EC3.2.1.4) belong to glycosyl hydrolase family 9 (GH9) [47] and are crucial for cell wall degradation and remodeling because they can cleave the internal β-1,4-glycosidic bond between two glucose moieties in the center of a polysaccharide chain. Natural substrates of most plant endo-1,4-β-glucanases are soluble cellulose derivatives such as carboxymethyl cellulose, noncrystalline phosphoric acid—swollen cellulose, and a variety of plant polysaccharides including xylans, β-(1,3)-(1,4)-glucans, and glucomannans [50, 55, 56]. The DNA polymorphism at *qHS1* that differentiates between Tachinagaha and TA-HS is a single-nucleotide substitution that changes serine 283 in TA-HS to isoleucine in Tachinagaha. This AA substitution was unique to permeable soybean cultivars and was located next to a highly conserved tryptophan residue in a substrate binding subsite for β-1,4-glucan chains. The AA substitution in Tachinagaha may therefore modify the enzyme function by interfering with its substrate affinity, resulting in a fragile cell wall structure susceptible to damage by desiccation during seed maturation in permeable soybean cultivars. Further evidence for the reduction or elimination of endo-1,4-β-glucanase function in permeable cultivars was provided by calcofluor white staining for β-1,4-glucan derivatives, which accumulated in the outer layer of palisade cells of TA-HS and of transgenic T2 plants containing *qHS1* from TA-HS but not in the permeable cultivar Tachinagaha or in the permeable cultivar Kariyutaka transformed with a control vector. Therefore, the difference in seed coat rigidity may reflect different levels of accumulation of carbohydrates containing β-1,4-glucan. By using berberine-aniline blue staining, Ma et al. (2004) found that an impermeable experimental line, OX951, displayed a prominent, yellowish-green cuticle under UV light, whereas permeable cultivars gave weak staining, suggesting a higher accumulation of callose (β-1,3-glucan) in the impermeable line [27].

The chemical composition of seed coats has been studied extensively in permeable cultivars and in impermeable experimental lines [26, 28, 30]. Mullin and Xu (2001) found that xylans in the hemicellulose of seed coat fractions, which are known to have low water affinity, correlated with the water uptake ratio and the occurrence of hard seeds [26]. On the other hand, Shao et al. (2007) found different monomer compositions of the cutin polymer in seed coats of permeable cultivars and the impermeable line OX951; the most notable difference was the relatively higher amount of hydroxylated components (especially 2-hydroxy- and ωs-hydroxy-fatty acids) in hard seeds from the impermeable line [28]. The presence of a greater proportion of hydroxylated fatty acids may provide a greater interconnectivity between monomers in the cutin of hard seeds. Shao et al. (2007) further suggested that a more extensive integration exists between cutin and carbohydrate monomers in the impermeable cultivar, which may give the surface material either more structural integrity or greater flexibility, making it less susceptible to cracking and therefore less permeable [28]. The endo-1,4-β-glucanases have a broad substrate specificity; for example, OsCe19 hydrolyzed 1,4-β-glycosyl linkages of carboxymethyl cellulose, phosphoric acid—swollen cellulose, β-(1,3),(1,4)-glucans, arabinoxylan, xylans, glucomannan, and cellooligosaccharides [56]. The findings obtained in the present study, coupled with those of previous studies [26, 27], suggest that a hard-seed allele at *qHS1* produces a functional endo-1,4-β-glucanase that causes β-1,4-glucan derivatives such as xyloglucan and β-(1,3),(1,4)-glucan to accumulate in the outer layer of palisade cells. These substances may make the seed coat more elastic and tolerant to desiccation-related stresses during maturation. A more detailed analysis of the biochemical function of GmGH9B8 is needed for better understanding of the mechanism by which hard seed coats are produced in soybean.

### The *qHS1* SNP Can Be a Useful Marker to Detect Genetically Controlled Stone Seeds

In addition to its adaptive role in wild plants, an impermeable seed coat provides some benefit to soybean cultivars in tropical and subtropical regions, where harvested seeds are stored under warm, humid weather conditions [15, 17]. Stone seeds, on the other hand, are often negative traits, particularly in soybeans intended for food processing [24–27]. Stone seeds are often produced under adverse environments during seed filling, such as low soil water availability [51–54] and high temperatures [57]. Our association test between the SNP at *qHS1* and seed coat permeability of soybean cultivars revealed that all of the impermeable seeds produced by cultivars of different origins possessed the impermeable allele at the SNP as either homozygotes or heterozygotes. In particular, the impermeable allele was observed at higher frequencies in cultivars from South and Southeast Asian countries than in cultivars from other regions (data not shown), suggesting that the prevalence of the impermeable allele in cultivars from these regions may reflect adaptation to high humidity and heat during seed filling and subsequent seed storage. The SNP may therefore represent a useful marker to trace whether the production of stone seeds is caused by *qHS1* or the environment, or possibly by one of the other QTLs for hard seededness yet to be characterized.

### Accession Numbers

Sequence data from this article were deposited in the GenBank/EMBL/DDBJ databases under accession numbers AB928216 and AB928217 for the cDNA sequences of the TA and TA-HS alleles at *qHS1*, and AB928218 for the genomic sequence of 6,273 bp used for transformation.

## Supporting Information

S1 Fig Expression profiles of 10 annotated genes in a 93-kb genomic region harboring *qHS1*. Transcriptional abundance was evaluated by a relative ratio to that of β-tubulin. Blue and red bars indicate transcriptional abundance in immature cotyledon and seed coat, respectively. Bars indicate the maximum errors of estimates at 95%. TA, Tachinagaha. TA-HS, a near-isogenic line of Tachinagaha for the hard-seed allele at *qHS1*.
(TIF)

S2 Fig cDNA sequences of *qHS1* for Tachinagaha and its impermeable NIL (TA-HS). Arrow, single nucleotide polymorphism.
(TIF)

S3 Fig Comparison of amino acid sequences among glycoside hydrolase family 9 enzymes. Predicted amino acid sequences of GmGH9B8-SS (soft seed, Tachinagaha) and GmGH9B8-HS (hard seed, TA-HS) were aligned with those of GH9 enzymes. OsCel9A, rice (*Oryza sativa* L.) endo-1,4-β-glucanase (Uniprot Q0JPJ1); PopCel1 and PopCel2, *Populus alba* endo-1,4-β-glucanases (Uniprot Q40763 and Q9XIY8, respectively); PttCel9A, a membrane-bound endo-1,4-β-glucanase (KOR) from *Populus tremula x tremuloides* (Δ1–105, Uniprot 6DMM4); and TfCel9A, *Thermobifida fusca* endo/exo-1,4-β-glucanase. Amino acids involved in substrate binding are shown with subsite numbers (+2 to ‒4). The catalytic residues are labeled “Cat”.
(TIF)

S4 Fig Structures and accumulation patterns of β-1,4-glucan derivatives of seed coats in transgenic plants. Structures of surfaces of seed coats in KA-GFP (A and B) and KA-qHS1 (C and D). Accumulation patterns of β-1,4-glucan derivatives in cross-section of seed coats from the dorsal side of seeds in KA-GFP (E and F) and KA-qHS1 (G and H). Arrows, cracks. Bars in A, C, F and H = 50μm; B and D = 10μm.
(TIF)

S1 Table Primer sequences for SSR markers used in fine-mapping of *qHS1*. (XLSX)

S2 Table Primers used for sequencing. (XLSX)

S3 Table Primers used for real-time PCR for 10 annotated genes in the 93-kb genomic region harboring *qHS1*. (XLSX)

S4 Table Annotation of predicted genes in a 93-kb genomic region harboring *qHS1* (Phytozome Glyma1.0). (XLSX)
