# Supplementary figures and images for: A Single-Nucleotide Polymorphism in an Endo-1,4-β-Glucanase Gene Controls Seed Coat Permeability in Soybean
Source: PLoS One. 2015 Jun 3;10(6):e0128527. doi: 10.1371/journal.pone.0128527 (PMC4454576; doi:10.1371/journal.pone.0128527)

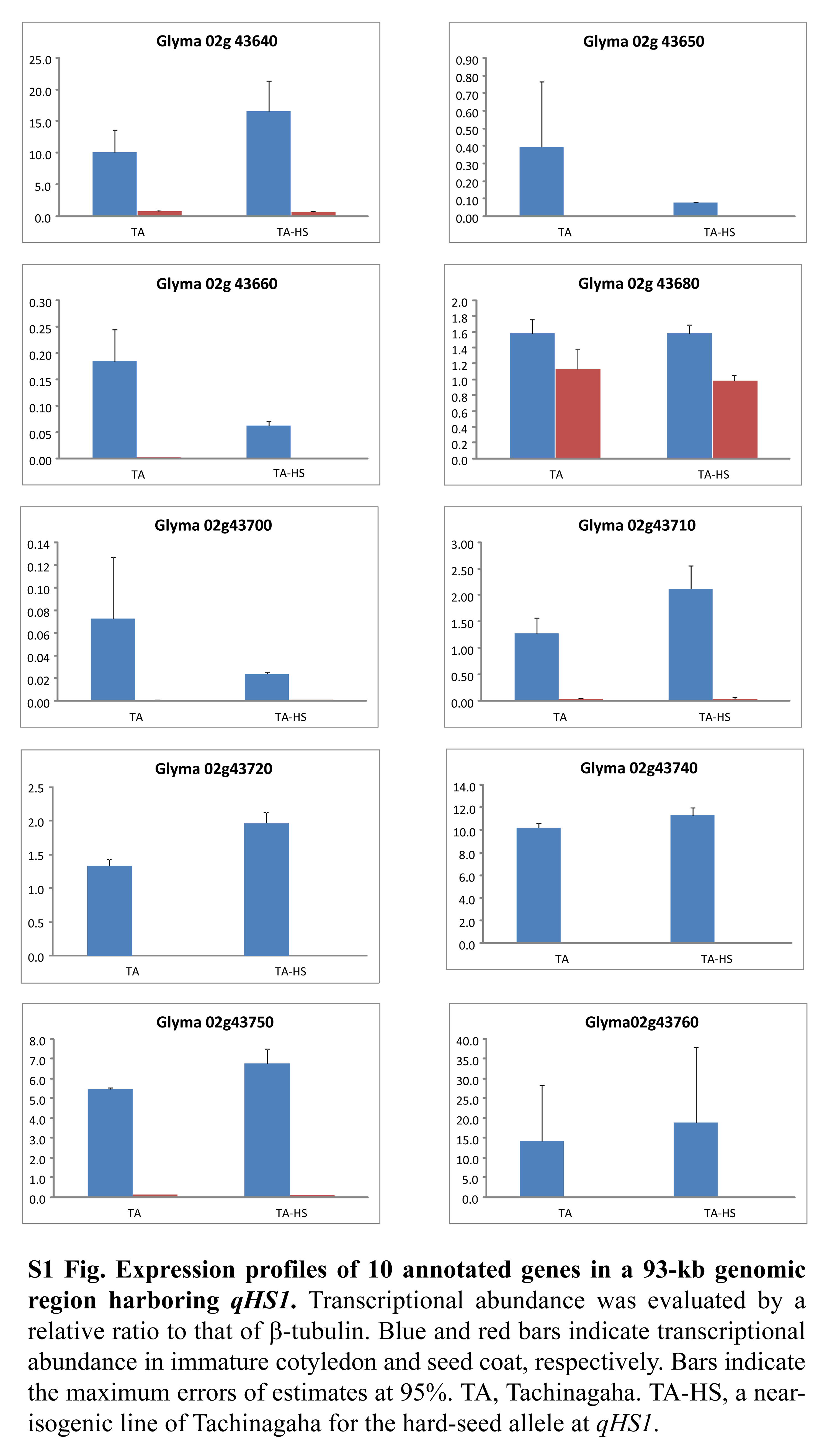

Supplement: S1 Fig — Transcriptional abundance was evaluated by a relative ratio to that of β-tubulin. Blue and red bars indicate transcriptional abundance in immature cotyledon and seed coat, respectively. Bars indicate the maximum errors of estimates at 95%. TA, Tachinagaha. TA-HS, a near-isogenic line of Tachinagaha for the hard-seed allele at qHS1. (TIF) [file pone.0128527.s001.tif]

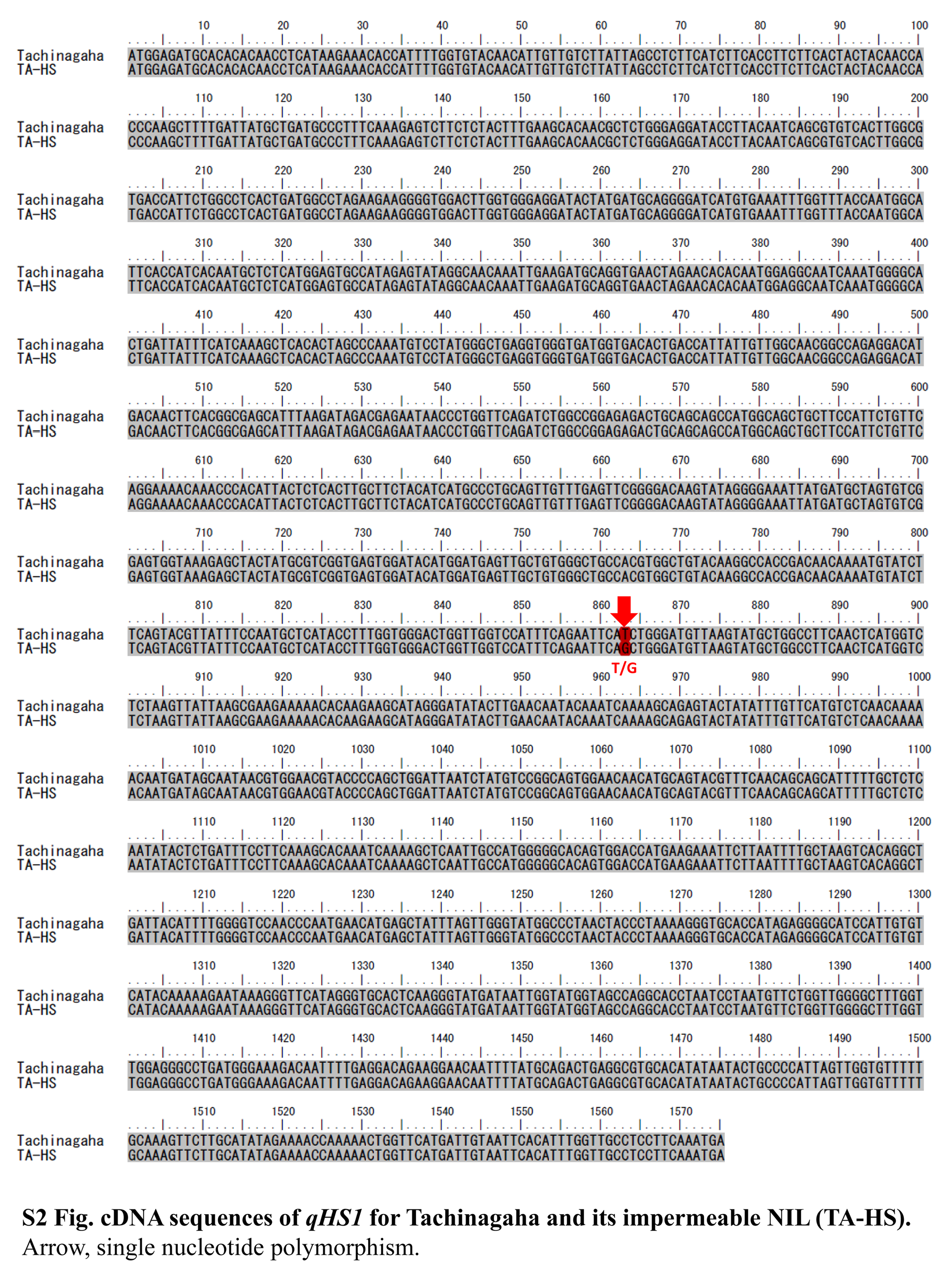

Supplement: S2 Fig — Arrow, single nucleotide polymorphism. (TIF) [file pone.0128527.s002.tif]

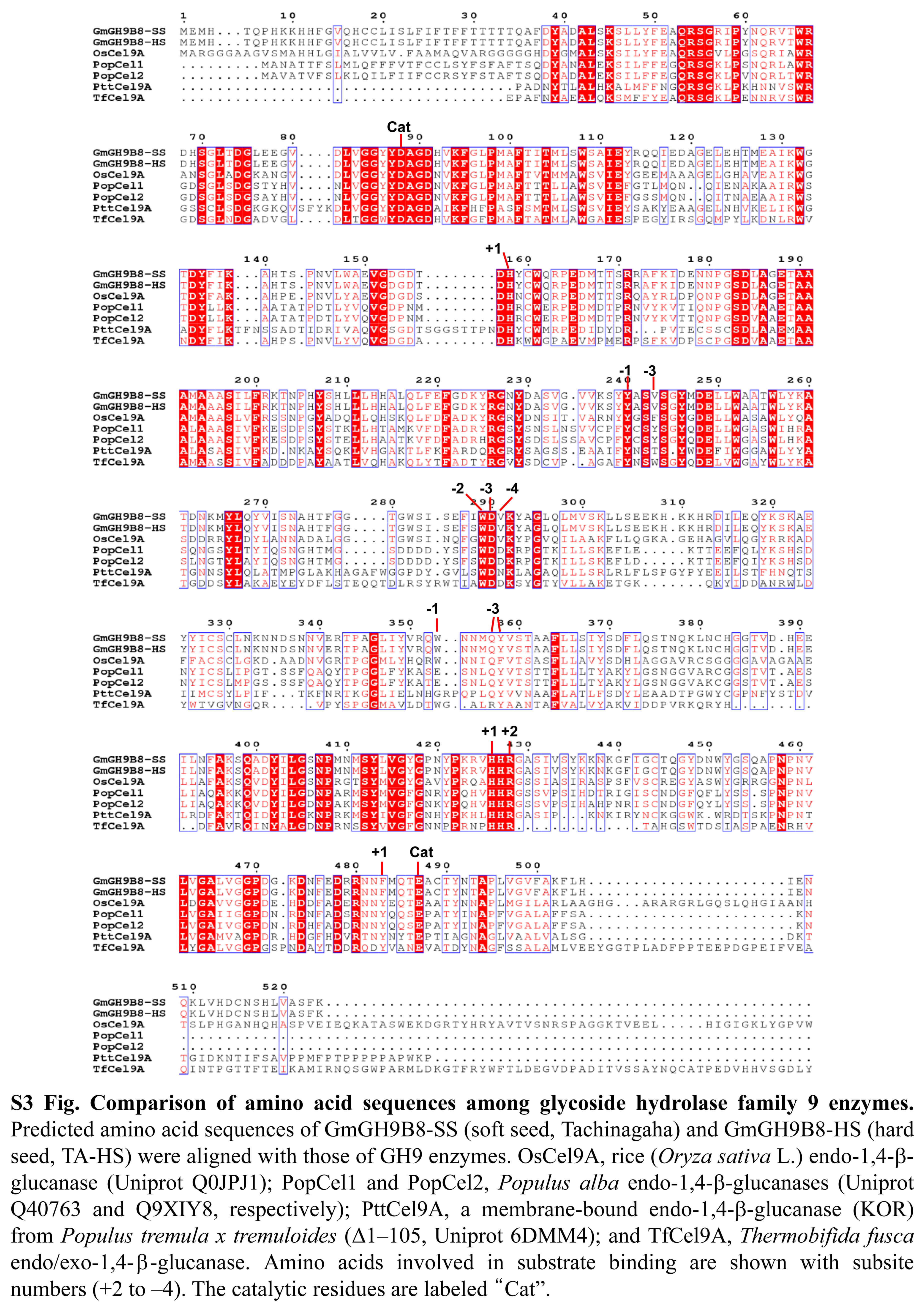

Supplement: S3 Fig — Predicted amino acid sequences of GmGH9B8-SS (soft seed, Tachinagaha) and GmGH9B8-HS (hard seed, TA-HS) were aligned with those of GH9 enzymes. OsCel9A, rice (Oryza sativa L.) endo-1,4-β-glucanase (Uniprot Q0JPJ1); PopCel1 and PopCel2, Populus alba endo-1,4-β-glucanases (Uniprot Q40763 and Q9XIY8, respectively); PttCel9A, a membrane-bound endo-1,4-β-glucanase (KOR) from Populus tremula x tremuloides (Δ1–105, Uniprot 6DMM4); and TfCel9A, Thermobifida fusca endo/exo-1,4-β-glucanase. Amino acids involved in substrate binding are shown with subsite numbers (+2 to ‒4). The catalytic residues are labeled “Cat”. (TIF) [file pone.0128527.s003.tif]

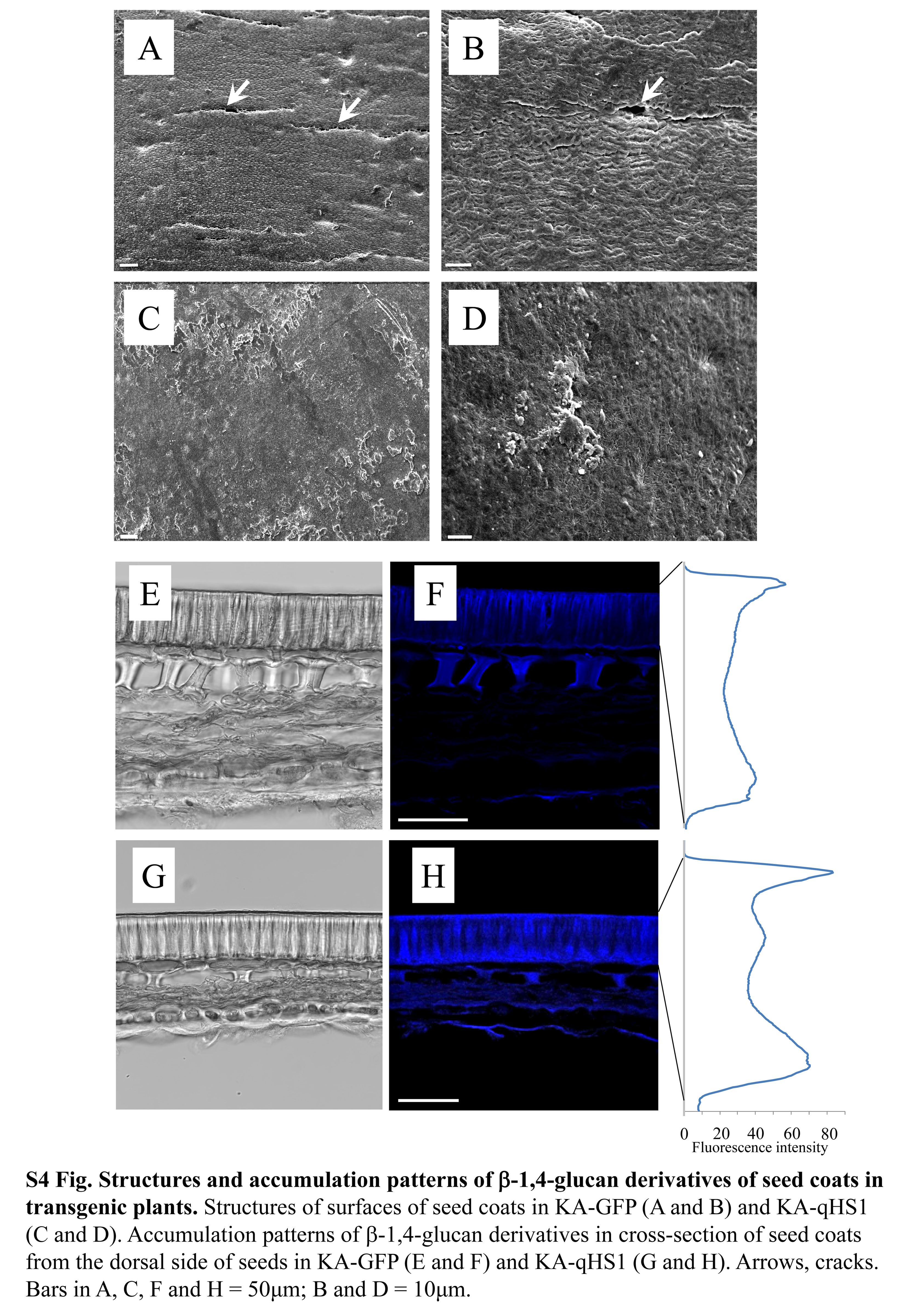

Supplement: S4 Fig — Structures of surfaces of seed coats in KA-GFP (A and B) and KA-qHS1 (C and D). Accumulation patterns of β-1,4-glucan derivatives in cross-section of seed coats from the dorsal side of seeds in KA-GFP (E and F) and KA-qHS1 (G and H). Arrows, cracks. Bars in A, C, F and H = 50μm; B and D = 10μm. (TIF) [file pone.0128527.s004.tif]
